# The Association Between the Processing of Binaural Temporal-Fine-Structure Information and Audiometric Threshold and Age: A Meta-Analysis

**DOI:** 10.1177/2331216518797259

**Published:** 2018-09-28

**Authors:** Christian Füllgrabe, Brian C. J. Moore

**Affiliations:** 1 Medical Research Council Institute of Hearing Research, School of Medicine, University of Nottingham, UK; 2 Hearing Sciences, Division of Clinical Neurosciences, School of Medicine, University of Nottingham, UK; 3 Department of Psychology, University of Cambridge, UK

**Keywords:** interaural phase, suprathreshold processing, aging, hearing loss, TFS-LF test

## Abstract

The ability to process binaural temporal fine structure (TFS) information, which influences the perception of speech in spatially distributed soundscapes, declines with increasing hearing loss and age. Because of the relatively small sample sizes used in previous studies, and the population-unrepresentative distribution of hearing loss and ages within study samples, it has been difficult to determine the relative and combined contributions of hearing loss and age. The aim of this study was to survey published and unpublished studies that assessed binaural TFS sensitivity using the TFS-low frequency (LF) test. Results from 19 studies were collated, yielding sample sizes of 147 to 648, depending on the test frequency. At least for the test frequency of 500 Hz, there were at least 67 listeners in each of four adult age groups and the distribution of audiometric thresholds at the test frequency within each group was similar to that for the population as a whole. Binaural TFS sensitivity declined with increasing age across the adult lifespan and with increasing hearing loss in old adulthood. For all test frequencies, both audiometric threshold and age were significantly negatively correlated with TFS-LF sensitivity (*r* ranging from −0.19 to −0.64) but the correlation was always significantly higher for age than for audiometric threshold. Regression analyses showed that the standardized regression coefficient was greater for age than for audiometric threshold, and that there was a significant interaction; the effect of increasing age among older listeners was greater when the hearing loss was ≥30 dB than when it was < 30 dB.

## Introduction

Changes in the temporal fine structure (TFS) of sounds at each ear and across the two ears are used for the perceptual analysis of complex auditory scenes and for sound localization (Moore, 2014). One example is speech perception in the presence of interfering background sounds, which has been shown to be associated with sensitivity to TFS (e.g., Füllgrabe, Moore, & Stone, 2015; Lopez-Poveda et al., 2017; Neher, Lunner, Hopkins, & Moore, 2012; Oberfeld & Klöckner-Nowotny, 2016; Strelcyk & Dau, 2009), although it is difficult to prove that the relationship is causal. There is increasing psychoacoustical evidence that TFS sensitivity tends to decline with increasing hearing loss (e.g., Füllgrabe & Moore, 2017; Gallun et al., 2014; Hopkins & Moore, 2011; King, Hopkins, & Plack, 2014; Pichora-Fuller & Schneider, 1992) and with increasing age (e.g., Füllgrabe, 2013; Füllgrabe et al., 2015; Füllgrabe, Sęk, & Moore, 2018; Grose & Mamo, 2010; Hopkins & Moore, 2011; King et al., 2014; Moore, Glasberg, Stoev, Füllgrabe, & Hopkins, 2012; Moore, Vickers, & Mehta, 2012; Ross, Fujioka, Tremblay, & Picton, 2007), even when the effect of the other variable is statistically or experimentally controlled. However, the relative influence of hearing loss and age on binaural sensitivity to TFS remains unclear. Hopkins and Moore (2011) found a significant group difference in binaural TFS sensitivity between young normal-hearing and older normal-hearing listeners but the difference between the latter group and older hearing-impaired listeners was not significant. Also, binaural TFS sensitivity has been found to be more highly correlated with age than with the audiometric threshold at the test frequency for listeners with normal or near-normal hearing (Füllgrabe, 2013; Füllgrabe et al., 2015; Füllgrabe et al., 2018; Moore, Glasberg, et al., 2012). Despite the fact that no statistical comparison of the correlation coefficients was conducted in those studies, it has been speculated that aging might have a stronger deleterious effect than hearing loss on binaural TFS sensitivity (Füllgrabe & Moore, 2017; Moore, 2016).

The results of King et al. (2014) do not support this speculation. They tested 46 listeners with a wide range of ages (20 to 83 years) and hearing thresholds (∼2 to 69 dB hearing level [HL]) and reported significant moderate correlations of similar size between thresholds for discriminating an interaural phase difference (IPD) and audiometric thresholds at the test frequency of 500 Hz (*r* = 0.42) and between IPD thresholds and age (*r* = 0.45). However, their sample of listeners was characterized by a similar incidence of hearing impairment (audiometric thresholds exceeding 20 dB HL) for the young (< 40 years; incidence: 37%) and older (> 60 years; incidence: 44%) listeners, and a weak nonsignificant correlation (*r* = 0.08) between age and the absolute threshold at low frequencies. These sample characteristics resulted from an explicit effort to recruit young listeners with sensorineural hearing loss (A. King, personal communication, February 15, 2018). While this allowed the authors to provide experimental evidence that both hearing loss and age affect binaural TFS sensitivity, it made it impossible to study the relative effects and interaction of hearing loss and age that would be expected across the adult lifespan in the general population. Indeed, low-frequency hearing loss is rather rare in the younger general population. For example, in the United Kingdom (UK), 90% of people aged 18 to 30 years have low-frequency audiometric thresholds (averaged over 250, 500, and 1000 Hz) better (i.e., lower) than 20 dB HL (Davis, 1995).

Clearly, the relative effects of hearing loss and age on TFS sensitivity measured in a given study will depend on the ranges of audiometric thresholds and ages in the study sample. In turn, these ranges might not be representative of the population as a whole. Only a study of TFS sensitivity for a large sample of the general population, in which hearing loss and age covary in a representative manner, can establish the independent and combined contribution of these variables throughout the lifespan. As such information is currently not available, and large-scale studies are costly and time-consuming, the aim of this study was to collate existing data from studies using listener samples with various audiometric and age distributions, in order to obtain a picture of the changes in binaural TFS sensitivity across the lifespan that is more representative than that which can be gleaned from individual published studies.

Binaural TFS sensitivity has been studied using a variety of behavioral tasks, such as the binaural masking level difference (BMLD; e.g., Neher, 2017; Pichora-Fuller & Schneider, 1991; Santurette & Dau, 2012; Strelcyk & Dau, 2009), interaural time difference (ITD) discrimination (e.g., Füllgrabe & Moore, 2014; Strouse, Ashmead, Ohde, & Grantham, 1998), and IPD discrimination (e.g., Füllgrabe, Harland, Sęk, & Moore, 2017; Ross et al., 2007; Strelcyk & Dau, 2009). One test that has been used in several recent studies is the TFS-LF test developed by Hopkins and Moore (2010) and implemented by Sęk and Moore (2012). Unlike some tests that have been described in the literature, such as BMLDs (Hafter & Carrier, 1970) or ITD discrimination tasks that require the listener to indicate whether a sound moved to the left or the right (Wright & Fitzgerald, 2001), practice effects for the TFS-LF test are small or absent (Hopkins & Moore, 2010), so there is no need for a protracted familiarization period prior to data collection. This might explain why this test has been frequently used since its publication, including in two large-scale studies (with *N* > 100; Füllgrabe, 2013; Rönnberg et al., 2016). Based on the frequency of its use and the existence of large data sets, it was deemed appropriate to focus on the TFS-LF test for the meta-analysis described here.

## Method

### Description of the TFS-LF Test

The TFS-LF test measures thresholds for detecting a change in IPD of bursts of low-frequency pure tones, presented via headphones. The envelopes of the tones are synchronous across the two ears, so there is an interaural disparity in the TFS only. We describe here the “standard” version of the TFS-LF test. Variants used in some of the studies included in the meta-analysis are described later.

The tones in each ear are presented at 30 dB sensation level (SL). A two-interval, two-alternative forced-choice task is used, with four successive tones in each interval (with the tone duration and the interstimulus interval being at least 400 and 200 ms, respectively). In one interval, selected randomly, the four tones all have the same IPD of 0°. In the other interval, the IPD alternates between 0° and ϕ in successive tones. Listeners with normal hearing and normal sensitivity to binaural TFS perceive pure tones with IPD = 0° as being close to the center of the head, while tones with a sufficiently large IPD are perceived as being lateralized toward one ear or the other, or as being more diffuse. Hence, the listeners are asked to indicate the interval in which the tones appear to change in some way, for example to move within the head. Correct-answer feedback is provided after each trial. The initial value of ϕ is usually set to the maximum value of 180° and ϕ is adaptively varied, using a two-down, one-up rule to converge at an estimate of the “threshold” corresponding to 71% correct. The threshold is computed as the geometric mean value of ϕ at the last six turnpoints.

If the adaptive procedure calls for the maximum value of ϕ (180°) twice before the second turnpoint or at all after the second turnpoint, the adaptive procedure is terminated and 40 further trials are presented with ϕ set to 180°; the number of correct responses is recorded to yield a percent-correct score.

### Derivation of a Performance Measure

The adaptive procedure of the TFS-LF test results in a threshold estimate in degrees, while the constant-stimulus procedure with ϕ set to 180° gives a percent-correct score. In this study, to compare results from the two procedures, the percent-correct scores from the constant-stimulus procedure were converted into values of the detectability index *d* ′, using the conversion table of Hacker and Ratcliff (1979). Thresholds from the adaptive procedure were converted into values of *d* ′ that would be obtained for ϕ = 180°, using the equation:

(1) d'=(0.78×180)/TFS-LFthreshold

where 0.78 is the *d* ′ value corresponding to the 71%-correct point on the psychometric function, the threshold tracked by the adaptive procedure (for further details, see Hopkins & Moore, 2010). Equation 1 is based on the finding that *d* ′ is proportional to ϕ, at least for *d* ′ values up to about 2 (Hafter & Carrier, 1972). Equation 1 can lead to very large values of *d* ′ when the TFS-LF threshold is small. These high values should not be taken literally; in practice, values of *d* ′ greater than about 3 are very hard to measure. The major advantage of using Equation 1 is that it allows a meaningful comparison of performance across the adaptive and constant-stimulus procedures; the larger the value of *d* ′, the better is performance.

### Selection of Studies for Inclusion

A search of published research papers, based on our knowledge of the literature, yielded 19 studies that used a version of the TFS-LF test to assess binaural TFS sensitivity. It was not possible to obtain data for three of these studies. Informal discussions with colleagues yielded an additional three (as yet) unpublished data sets (Barry, unpublished; Bramsløw, Eneroth, Lunner, & Schulte, 2012; Rost, Ellermeier, Kattner, & Oberfeld, 2018). Thus, a total of 19 studies were entered into the meta-analysis and are referenced in Table 1 in alphabetical order. For studies using distinct samples of listeners (e.g., young vs. older), each sample is described separately. The age range, the test frequencies, the range of audiometric thresholds at each test frequency, and the size of each sample are indicated. In some cases, the latter does not match the number of listeners in the original data set, as results from listeners with an interaural difference in audiometric threshold exceeding 15 dB at the test frequency were not included in the meta-analysis. This was done to follow the audiometric inclusion criterion of symmetric hearing sensitivity used in most (but not all) previous studies investigating binaural TFS sensitivity. Neher (2017) reported that there were no differences in binaural TFS sensitivity between listeners with symmetric and asymmetric hearing losses at low frequencies. However, his study assessed BMLDs (and not IPD discrimination) and hence it was deemed prudent to exclude the few asymmetric cases observed in some data sets from the meta-analysis.

**Table 1.** Listener-Sample Details (Age Range, Audiometric Range, and Number of Listeners, *N*) and Test-Tone Frequencies From Studies Included in the Meta-Analysis.

Study	Age range (years)	Audiometric range (dB HL)	*N*	Test frequency (Hz)			
Barry (unpublished)	6 to 12	−3 to 20	38		500		
	20 to 38	−5 to 18	25		500		
Bramsløw et al. (2012)	24 to 83	25 to 73	22	250	500	750	
Füllgrabe (2013)	19 to 90	−8 to 20	112		500		850
Füllgrabe et al. (2015)	18 to 27	−1 to 10	8		500	750	
	60 to 79	−10 to 11	21		500	750	
Füllgrabe and Moore (2017)	63 to 83	3 to 20	12	250			
Füllgrabe et al. (2017)	19 to 25	−5 to 8	9	250	500	750	
	47 to 84	3 to 18	23			750	
Hopkins and Moore (2011)	20 to 35	−9 to 8	10	250	500	750	
	62 to 69	−3 to 11	6	250	500	750	
	25 to 82	−4 to 55	24	250	500	750	
Lőcsei et al. (2016)	20 to 29	−5 to 15	10	250			
	55 to 84	5 to 28	19	250			
Moore, Glasberg, et al. (2012)	61 to 83	1 to 44	39		500	750	
Moore et al. (2012)	22 to 61	1 to 23	35		500		850
Moore and Sęk (2016)	56 to 87	13 to 58	22		500		
Neher et al. (2012)	28 to 75	22 to 55	17	250	500	750	
Oberfeld and Klöckner-Nowotney (2016) ^a^	18 to 30	−4 to 9	50		500		
Perez et al. (2014)	54 to 85	13 to 53	51		500		
Rönnberg et al. (2016)	27 to 80	0 to 65	196	250			
Rost et al. (2018)^a^	17 to 37	0 to 15	67		500		
Sharma et al. (2014) ^b^	11 to 15	5 to 14	15		500		
Whitmer and Akeroyd (2013)	23 to 75	−3 to 108	44		500		
Whitmer et al. (2014)	26 to 79	−5 to 68	33		500		

a Audiometric thresholds were assessed using Bekesy tracking and 1/3-octave noise bands.

b This study tested normal-hearing children with and without listening difficulties. Only data from the children with no listening difficulties were included here. Audiometric thresholds for individual frequencies were not available, so the pure-tone average across 500, 1000, and 2000 Hz was used.

### Characteristics of the Study Samples

Most of the data were for adult listeners, aged between 18 and 90 years; only two studies investigated children (aged 6 to 15 years; *N* = 53). For all samples combined, the audiometric threshold at the test frequency varied from “normal” to “profoundly impaired” (from −10 to 108 dB HL; British Society of Audiology, 2011). Four tone frequencies were used: 250, 500, 750, and 850 Hz. The largest data set was for 500-Hz tones (*N* = 648), followed by 250-Hz tones (*N* = 325), 750-Hz tones (*N* = 178), and 850-Hz tones (*N* = 147).

**Figure 1.**
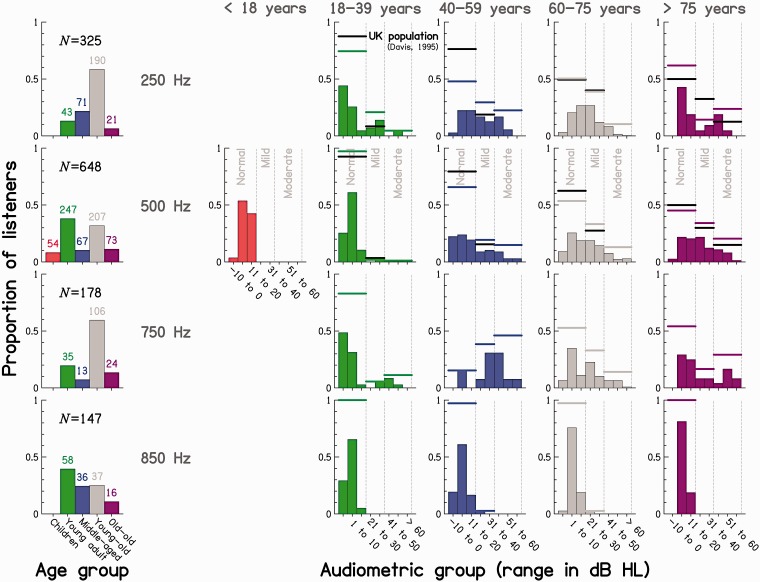
Distribution in terms of proportions (bars) and numbers (colored numbers) of listeners entered into the meta-analysis. *First column:* Based on their age, listeners were categorized into five age groups: “children” (< 18 years), “young adults” (18 to 39 years), “middle-aged adults” (40 to 59 years), “young-old adults” (60 to 75 years), and “old-old adults” (> 75 years). *Columns 2 to 6*: Based on their audiometric thresholds at the test frequencies, listeners for each age group were categorized into eight bins between −10 and > 60 dB HL (see colored bars) or into the three lowest hearing-loss categories (see colored horizontal lines), as defined by the British Society of Audiology (2011): “normal hearing” (−10 to 20 dB HL), “mild hearing loss” (21 to 40 dB HL), and “moderate hearing loss” (41 to 70 dB HL). As there were only three cases of hearing losses greater than 70 dB HL, these listeners were included in the “moderate” category. For comparison, distributions of audiometric thresholds for the general population are indicated by the thin black horizontal lines (Davis, 1995). Each row shows results for one of the test frequencies.

To illustrate how the listeners were distributed across ages and audiometric thresholds, the left-most column of Figure 1 shows the proportion and number of listeners in each of five age groups (“children”, “young adults”, “middle-aged adults”, “young-old adults”, and “old-old adults”) for each of the four test frequencies; the remaining columns show the proportion of listeners in each of eight audiometric categories (“−10 to 0”, “1 to 10”,…, “> 60”) for each of the five age groups for the given test frequency. The colored thick lines indicate proportions of listeners in terms of broader clinically used hearing-loss categories (“normal hearing”, “mild hearing loss”, and “moderate hearing loss”), here based on the audiometric ranges specified by the British Society of Audiology (2011).

Despite our pooling of data across multiple studies from different research groups, resulting in fairly large data sets, the different age groups were not uniformly represented, with either the young or the young-old adults always representing the largest listener group, and these two groups combined constituting at least 65% of the listeners tested at any given tone frequency. In a few cases, the sample size was relatively small, below 20 (see middle-aged listeners at 750 Hz and old-old listeners at 850 Hz). This bias in the age distribution probably reflects the consequences of the goal of many of the studies included in the meta-analysis, which was to study age-group differences in the absence of hearing loss. The bias may also reflect the availability of research participants (generally sampled from among the student population and early retirees).

For children and young adults, most audiometric thresholds at all test frequencies fell within the normal range, while, for older adults, they tended to be more evenly distributed across the three broad hearing-loss categories. An exception is the distribution of audiometric thresholds at 850 Hz; here, almost all listeners, independent of age group, had normal hearing. To indicate how well the distributions of audiometric thresholds are representative of those found in the general population, data from the population-representative “National Study of Hearing” (Davis, 1995), based on age groups and audiometric categories roughly equivalent to those used in here, are plotted in Figure 1 as thick black horizontal lines for the 250-Hz and 500-Hz test frequencies (no reference data were available for the two other frequencies). The distributions of audiometric thresholds in our meta-analysis are not markedly different from those for the UK population, especially at 500 Hz. However, for the middle-aged group at 250 Hz, the current data sets have greater proportions of mild and moderate losses than for the population data.

### Methods Used in the Study Sample

Most of the studies included in the meta-analysis conformed to the standard experimental procedure (such as the use of a two-down, one-up stepping rule, tracking the 71% correct point) and stimulus parameters (such as the duration of the stimuli and interstimulus intervals), as described in the original article describing the TFS-LF test (Hopkins & Moore, 2010), but there were a few exceptions. Perez, McCormack, and Edmonds (2014) and Rönnberg et al. (2016) used, respectively, a lower (10 dB SL) and higher (40 dB SL) presentation level than the 30 dB SL recommended by Hopkins and Moore (2010), who showed that similar results are obtained for SLs between 30 and 50 dB but performance worsens for a lower SL of 20 dB. Hence, the performance reported by Perez et al. might be worse than that observed in other studies for listeners with comparable ages and degrees of hearing loss. All relevant analyses were computed with and without this data set (*N* = 51), representing 8% of the data for the 500-Hz test tones. As the two sets of results were always very similar, only the results for all data are reported here.

Four studies (Oberfeld & Klöckner-Nowotny, 2016; Rost et al., 2018; Whitmer & Akeroyd, 2013; Whitmer, Seeber, & Akeroyd, 2014) used a three-down, one-up rule, thereby tracking a higher performance level (i.e., 79% correct) than for the other studies. As described in the *Derivation of a Performance Measure* section, the raw data produced by the TFS-LF test (thresholds expressed in degrees or percent-correct scores for the constant-stimulus procedure) were transformed into values of *d* ′. The higher *d* ′ value of 1.14 corresponding to 79% correct was used for the four studies that used the three-down, one-up rule, thus allowing comparison across data sets obtained using different stepping rules. For listeners who did not score above chance in the constant-stimulus procedure, *d* ′ was set to 0 (this happened only in 13 cases; Whitmer & Akeroyd, 2013; Whitmer et al., 2014).

### Data Analysis

To analyze the data, separate bivariate and partial correlational analyses were conducted for each test frequency to assess the relationship between *d ′* scores and audiometric thresholds on the one hand and between *d* ′ scores and age on the other. As we were testing the hypotheses that *d* ′ scores would worsen with increasing hearing loss and increasing age, one-tailed tests were used to assess the significance of the correlations. The significance of the differences between pairs of correlation values was assessed using two-tailed tests. Subsequently, multiple-regression analyses were run for the different test frequencies to study the relative contributions of audiometric threshold and age, as well as their interaction, to binaural TFS sensitivity. Given the limited availability of TFS-LF data for children and the possibility of developmental changes in basic auditory processing during childhood (Moore, Cowan, Riley, Edmondson-Jones, & Ferguson, 2011), it was decided to limit the correlation and regression analyses to results from adults (≥18 years).

## Results

**Figure 2.**
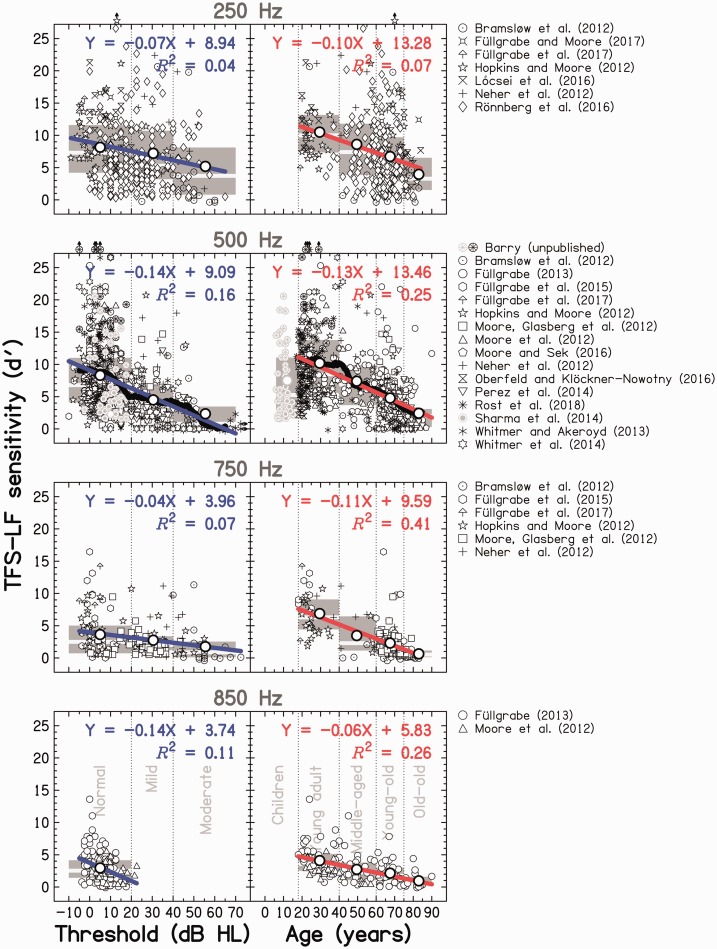
Binaural TFS sensitivity measured by the TFS-LF test and expressed as the sensitivity index *d*′. Individual data from several studies (see different symbols in the key at the right) using different low-frequency pure tones (see rows) are plotted as a function of the listeners’ audiometric thresholds (left column) and ages (right column). Vertical dotted lines in the left column show the boundaries of the hearing-loss catergories proposed by the British Society of Audiology (2011), while those in the right column show the boundaries of the five age-group categories. Light-gray and black symbols denote data from children and adults, respectively. Data points from adult listeners that fell outside the range of audiometric thresholds (*N* = 2) or the range of *d*′ scores (*N* = 5) used in the panels are denoted by symbols with arrows. The dark-gray-shaded areas and white thick horizontal lines represent the IQR and median, respectively, for each of the three audiometric groups and the five age groups. The large open circles, plotted at the center of the audiometric or age group, represent the mean. In each panel, the thick colored line is a regression line fitted to the individual data. The corresponding regression equation and *R*^2^ value are indicated. For the 500-Hz condition (second row from the top), the thick black line shows the running average, computed, in the left panel, as the arithmetic mean over an 11-dB-wide symmetric rectangular window centered on audiometric thresholds between −5 and 65 dB HL (in 1-dB steps), and, in the right panel, as the running average over an 11-year-wide symmetric rectangular window centered on ages between 23 and 85 years (in 1-year steps).

An overview of the results is given in Figure 2. For the single tone frequency for which data for children were available, all children were able to perform the TFS-LF test. On average, TFS-LF sensitivity for children (mean *d* ′ score = 7.5) was lower (worse) than that for young adults (mean *d* ′ score = 10.2) but on a par with that for middle-aged adults (mean *d* ′ score = 7.5). It is noteworthy that the two studies investigating adjacent age ranges for young listeners gave clearly different results: while performance for the younger children (*N* = 38; 6 to 12 years) spanned a wide range (*d* ′ scores ranged from 1.6 to 28.5, similar to the range for young adults), *d* ′ scores for the relatively small group of older children (*N* = 15; 11 to 15 years) consistently fell below 8. It is not clear whether this discrepancy was because of age differences, a sampling bias, or study differences. For the entire group of children aged below 18 years, TFS-LF sensitivity was not significantly correlated with audiometric thresholds (*r* = −0.04, *p* = 0.45) or with age (*r* = −0.32, *p* = 0.12).

Consistent with previous investigations assessing TFS-LF thresholds for different tone frequencies using small-sized samples of adult listeners (Füllgrabe et al., 2015; Hopkins & Moore, 2010, 2011), the highest and average TFS-LF sensitivity declined with increasing tone frequency (compare across rows).

### Relationship of *d*′ Values With Audiometric Threshold

For the three highest test frequencies (see the three lower panels of the left column of Figure 2), the scatter plots relating *d* ′ scores to audiometric threshold were roughly triangular in shape. For normal-hearing listeners (with audiometric thresholds ≤ 20 dB HL), *d* ′ scores spanned the range from the highest values observed in the meta-analysis to near or at 0. The highest and average *d* ′ scores declined with increasing audiometric threshold, while *d* ′ scores near 0 occurred across the whole range of audiometric thresholds, from moderately impaired to normal hearing. At 250 Hz, the scatter plot had a round shape and fewer listeners were unable to perform the test.

Linear regression lines were fitted to the individual *d* ′ scores as a function of audiometric threshold at the test frequency and are shown as the thick blue lines in the left column of Figure 2. The equation for the regression line and the percent variance explained are shown in each panel. Also, a running average of the data at 500 Hz (which had the highest *N*) was computed using an 11-year rectangular time window (see thick black line on the left of Figure 2), to assess whether there were deviations from linearity. This window duration was chosen as it resulted in at least seven data points within each window, hence smoothing the effect of individual variations in performance. The running average closely followed the regression line for that frequency. The strength of the association between *d* ′ scores and audiometric thresholds for each of the four test frequencies is shown as Pearson correlations in column 2 of Table 2. For the test frequencies for which the listeners’ audiometric thresholds covered a wide range (i.e., 250, 500, and 750 Hz), the bivariate correlation coefficients were −0.19, −0.40, and −0.27, respectively (all *p* < 0.001). At 850 Hz, audiometric thresholds only varied over the normal hearing range (i.e., between −10 and 20 dB HL), but the correlation of −0.34 was significant (*p* < 0.001). Thus, depending on the test frequency, between 4% and 16% of the variance in *d* ′ scores was accounted for by audiometric thresholds. The correlations between *d* ′ scores and audiometric threshold with the effect of age partialled out are shown in column 3 of Table 2. All partial correlations were significant (all *p* ≤ 0.007) except for the partial correlation at 750 Hz which just failed to reach significance (*p* = 0.052). All partial correlations were consistently lower than the bivariate correlations (by a factor of 1.4 to 2.2), suggesting that part of the effect of audiometric threshold could be explained by the association between audiometric threshold and age. The partial correlations accounted only for 2% to 5% of the variance in the *d* ′ scores.

**Table 2.** Bivariate and Partial Pearson Correlations (With Associated One-Tailed Significance Levels Within Parentheses) Between TFS-LF Thresholds (Expressed as *d*′ Scores) on the One Hand and Audiometric Threshold (in Hz; Rows 2 and 3) and Age (in Years; Rows 4 and 5) on the Other, for Each of the Four Test Frequencies.

Test frequency	Audiometric threshold		Age	
	Bivariate	Partial	Bivariate	Partial
250 Hz	−.191 (<.001)	−.135 (.007)	−.262 (<.001)	−.226 (<.001)
500 Hz	−.400 (<.001)	−.192 (<.001)	−.502 (<.001)	−.377 (<.001)
750 Hz	−.266 (<.001)	−.123 (.052)	−.642 (<.001)	−.614 (<.001)
850 Hz	−.336 (<.001)	−.220 (.004)	−.509 (<.001)	−.453 (<.001)

*Note.* For the partial correlations, the effect of the other variable (age in the case of audiometric threshold, and vice versa) was partialled out. No correction for multiple comparisons was applied.

While the absolute amount of variability of *d* ′ scores (in terms of their range or interquartile range, IQR) generally decreased with increasing audiometric loss, the coefficient of variation ([SD/mean] × 100), a measure of relative variability, actually increased across the three audiometric groups (“normal hearing”, “mild hearing loss”, and “moderate hearing loss”, as defined by the British Society of Audiology, 2011), being 71%, 78%, and 99% at 250 Hz, 71%, 91%, and 156% at 500 Hz, and 98%, 91%, and 145% at 750 Hz, respectively. Comparing the coefficients of variation across audiometric groups, using Levene’s *F* test, indicated a significant effect at 250 Hz [*F*_(2,322)_ = 5.73, *p* = 0.004] and at 500 Hz [*F*_(2,591)_ = 22.51, *p* < 0.001], but not at 750 Hz [*F*_(2,175)_ = 2.54, *p* = 0.082]. Subsequent uncorrected one-tailed *t* tests on the data for the two lower test frequencies revealed that there were significant differences between all groups (all *p* < 0.05).

### Relationship of *d*′ Values With Age

Performance on the TFS-LF test, in terms of highest individual and mean scores, worsened with increasing age (right column of Figure 2). Linear regression lines were fitted to the individual *d* ′ scores as a function of age, and are shown as the thick red lines. The equation for the regression line and percent variance explained are shown in each panel. A running average was computed for the 500-Hz test frequency using an 11-year rectangular time window (thick black line on the right of Figure 2), resulting in at least 24 data points within each window. This closely followed the regression line, except between the ages of 30 and 45 years: average scores remained roughly constant up to about 40 years before declining more rapidly than the regression line between 40 and 45 years. The bivariate correlation coefficients (see column 4 of Table 2) were −0.26, −0.50, −0.64, and −0.51 for the four test-tone frequencies in increasing order, respectively (all *p* < 0.001), indicating that age explained between 7% and 41% of the variance in *d* ′ scores. The correlations between *d* ′ scores and age with the effect of audiometric threshold partialled out are shown in column 5 of Table 2. The partial correlations were only slightly lower than the bivariate correlations (by a factor of 1.0 to 1.3), and all partial correlations were highly significant (all *p* < 0.001), suggesting that most of the effect of age on *d* ′ scores cannot be attributed to the correlation between age and absolute threshold. The partial correlations explained 5% to 23% of the variance in the *d* ′ scores.

The range and IQR of the *d* ′ scores generally decreased with increasing age, while the coefficient of variation increased across the four age groups, being 41%, 71%, 91%, and 81% at 250 Hz, 60%, 71%, 81%, and 114% at 500 Hz, 47%, 93%, 106%, and 109% at 750 Hz, and 54%, 81%, 77%, and 128% at 850 Hz. Based on Levene’s *F* test, the coefficients of variation across age groups differed significantly across age groups at 250 Hz [*F*_(3,321)_ = 4.09, *p* = 0.007] at 500 Hz[*F*_(2,590)_ = 7.55, *p* < 0.001], and at 850 Hz [*F*_(3,143)_ = 5.75, *p* = 0.001], but not at 750 Hz [*F*_(3,174)_ = 1.89, *p* = 0.133]. Subsequent uncorrected one-tailed *t* tests on the data for the three frequencies for which a significant age-group effect was observed revealed that the variability for the young adults differed significantly (all *p* < 0.05) from that for all other age groups at 250 Hz, from that for the young-old and old-old adults at 500 Hz, and from that for the middle-aged and old-old adults at 850 Hz.

### Comparing Relationships

Although changes in TFS-LF sensitivity followed the same general trends with increasing audiometric threshold and increasing age, there was one noticeable difference. While very low *d* ′ scores occurred across the whole range of audiometric thresholds, *d* ′ scores for listeners up to midlife (∼40 to 45 years), almost all of whom had audiometric thresholds within the normal range at the test frequency (see column 3 in Figure 1), only very exceptionally approached 0 and generally remained above 5, 3, 3, and 1 for the test frequencies 250, 500, 750, and 850 Hz, respectively. Thus, younger listeners were usually able to perform the TFS-LF task. However, it appears that a substantial or complete loss of sensitivity to binaural TFS can occur with increasing age above 45 years even when the audiometric threshold at the test frequency remains within the normal range.

The correlation between *d* ′ score and age was always higher than the correlation between *d* ′ score and audiometric threshold (compare columns 2 and 4 in Table 2). Based on the test described by Lee and Preacher (2013), which assesses the significance of the difference between two correlations with one variable in common (*d* ′ score in this case; Steiger, 1980) using Fisher’s *r*-to-*z* transform, the differences were significant at 500 Hz (*p* = 0.0034), 750 Hz (*p* < 0.0001), and 850 Hz (*p* = 0.041), but not at 250 Hz (*p* = 0.28). Columns 3 and 5 of Table 2 show the partial correlations for each variable (audiometric threshold or age) with the effect of the second variable partialled out. At each test frequency, the partial correlation between *d* ′ score and age was higher than that between *d* ′ score and audiometric threshold. Using Fisher’s *r*-to-*z* transform, the differences in partial correlations (two-tailed test) were significant at 500 Hz (*p* = 0.007), 750 Hz (*p* < 0.001), and 850 Hz (*p* = 0.024), but not at 250 Hz (*p* = 0.234). Overall, these results suggest that, within the population sampled, *d* ′ scores were more related to age than to audiometric thresholds.

To predict performance on the TFS-LF test, separate multiple linear regression analyses were conducted, one for each of the four test frequencies, using the “enter” method, in which both predictors (age and audiometric threshold) are simultaneously entered. For all test frequencies, the two-predictor model was significant (all *p* < 0.001). Details of the regression analyses are shown in Table 3. When combined, age and audiometric thresholds accounted for between 8% and 42% of the variation in *d* ′ scores. Regression coefficients for both predictors were significantly different from 0 (all *p* ≤ 0.015), except for audiometric threshold at 750 Hz (*p* = 0.10). In all analyses, the standardized regression coefficient for age was larger than that for audiometric threshold.

**Table 3.** Results of the Two-Predictor (Age and Audiometric Threshold) Multiple Linear Regression Model for Each Test Frequency.

Test frequency	*R*	Adjusted *R*^2^	*β* (*p* value)	Standardized *β*
250 Hz	.293	.080	−.086 (<.001)	−.229
			−.050 (.015)	−.135
500 Hz	.528	.277	−.104 (<.001)	−.402
			−.067 (<.001)	−.193
750 Hz	.649	.415	−.105 (<.001)	−.615
			−.016 (.104)	−.098
850 Hz	.543	.286	−.052 (<.001)	−.449
			−.085 (.008)	−.199

*Note. R =* Multiple correlation coefficient; adjusted *R*^2 ^= adjusted variance explained; *β* (*p* value) = regression coefficients for age and audiometric threshold (top and bottom entries for each frequency) with associated significance levels within parentheses; standardized *β* = standardized regression coefficients.

**Figure 3.**
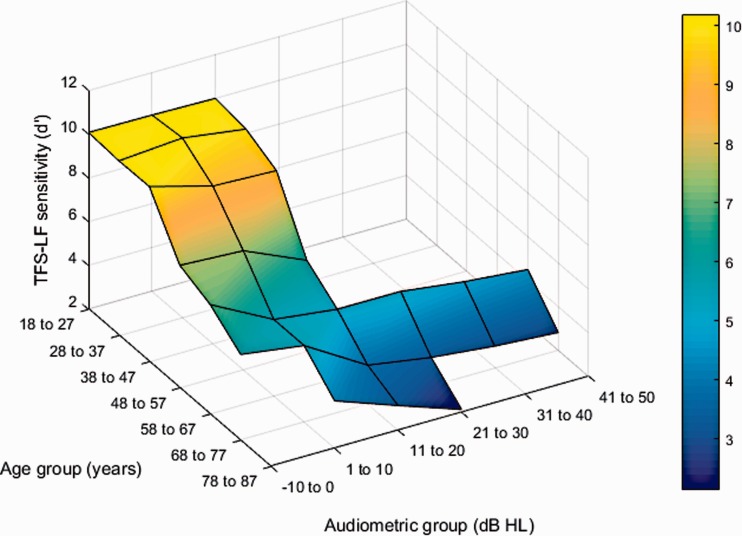
Changes in binaural TFS sensitivity at 500 Hz as a function of hearing loss and age. Performance on the TFS-LF test in terms of mean *d*′ scores (*z* axis) is plotted for 10-year-wide age groups (*x* axis) and roughly 10-dB-wide audiometric-threshold groups (*y* axis). Results are only shown for audiometric-threshold group × age group combinations for which at least five *d*′ scores were available. To smooth the data, the “true” value for each point was replaced by the mean of the value for that point and the values for the immediately adjacent points (higher and lower ages and greater and smaller audiometric losses).

As the sample size was largest for the test frequency of 500 Hz, all adult age groups were reasonably well represented for that frequency, and the distribution of audiometric thresholds for each age group followed that of the general population (see the second row in Figure 1), this data set was further analyzed. Figure 3 shows a three-dimensional representation of mean *d* ′ scores as a function of audiometric threshold and age group. Binaural TFS sensitivity worsened from young to old-old adulthood, while it was additionally affected by audiometric threshold in old adulthood.

To test for an interaction between age and audiometric threshold, two additional multiple linear regression analyses were performed, using age, audiometric threshold, and their interaction term (age × audiometric threshold) as predictors. For better interpretability of the regression coefficients, both predictors were centered (by subtracting a constant value from each datum point of a given predictor) prior to running the regression analysis (Dalal & Zickar, 2012; Jaccard, Wan, & Turrisi, 1990). Centering does not affect the multiple correlation coefficient, the significance level of the model, or the regression coefficient of the interaction term. However, a desirable consequence of this transformation is that the regression coefficient for the second predictor reflects its influence on the dependent variable at the chosen value of the first predictor, and not when the latter equals zero (which in the case of the second predictor audiometric threshold would correspond to a meaningless “age zero” for the first predictor). Using more than one centering allows estimation of the change in *d* ′ scores as a function of one predictor (e.g., audiometric threshold) for different values of the other predictor (e.g., the effect of audiometric threshold on *d* ′ scores in young and old adulthood). Such a transformation was not necessary for the previous additive regression models, which tested only the main effects of age and audiometric threshold, as in those cases, the regression coefficient for one predictor reflects the influence of that predictor on the dependent variable across the values of the other predictor.

The first analysis used the age and audiometric-threshold means (25 years and 5.3 dB HL, respectively) for the adult listeners aged below 40 years, for whom audiometric variability in the normal range did not seem to affect binaural TFS sensitivity, to center the predictors. Incorporating the interaction between age and audiometric threshold yielded a significant three-predictor model that explained a similar amount of variation in *d* ′ scores (adjusted *R*^2 ^= 0.281; *p* < 0.001) to that for the two-predictor model (adjusted *R*^2 ^= 0.277). The regression coefficients for age (*β* = −0.098; *p* < 0.001) and for the age × audiometric threshold interaction (*β* = −0.002; *p* = 0.042) were both significant, but the regression coefficient for audiometric threshold was not (*β* = −0.009, *p* = 0.785). The corresponding standardized regression coefficients were −0.378, −0.025, and −0.196, for age, audiometric threshold, and their interaction, respectively. The second analysis used the age and audiometric-threshold means for the adult listeners aged 60 years and above (70.7 years and 22.4 dB HL, respectively) to center the predictors. This time, all three regression coefficients were significant (for age: *β* = −0.125; *p* < 0.001; for audiometric threshold: *β* = −0.081; *p* < 0.001; for age × audiometric threshold: *β* = −0.002; *p* < 0.042). The corresponding standardized regression coefficients were −0.483, −0.235, and −0.129, for age, audiometric threshold, and their interaction, respectively. The interaction probably reflects the observation that, among listeners aged 58 years and above, the effect of increasing age is greater when the hearing loss is greater than 30 dB than when it is less than 30 dB (see Figure 3).

Taken together, the regression model incorporating an interaction term only marginally improved the amount of variability in *d* ′ scores explained, but it did reveal a significant interaction component. The significance of the regression coefficients and the size of the standardized regression coefficients depended on the group means used to center the data.

## Discussion

Most previous studies of the effects of audiometric threshold and/or age on binaural TFS sensitivity have used relatively small numbers of listeners, which would have limited the ability to detect weak associations. The median number of listeners in the studies used in our meta-analysis was 34; the average was higher at 48 because of the two larger-scale studies of Füllgrabe (2013) and Rönnberg et al. (2016). With such a small median sample size, the correlation between *d* ′ scores and audiometric threshold or age has to be at least 0.34 to yield a 95% confidence interval that does not include 0. The main motivation for the present study was to assess the effects of audiometric threshold and age on binaural sensitivity to TFS, using a large sample of listeners, who were audiometrically representative of the general population, and with a reasonably large number of listeners within each age group. While listeners aged 18 to 39 years and 60 to 75 years were somewhat overrepresented relative to the other age groups, there were substantial numbers of listeners in each adult age group, at least for the 500-Hz test frequency (for which the minimum number of listeners in any given adult age group was 67). Also, for this frequency, the distribution of audiometric thresholds in our sample followed that of the general population fairly closely (see the second row in Figure 1). Therefore, the results presented here probably are reasonably representative of those that would be found for the general population.

The results of our meta-analysis confirm previous reports that both audiometric threshold and age are correlated with the ability of adult listeners to process binaural TFS information. Our results showed that binaural TFS sensitivity was more strongly associated with age than with the audiometric threshold at the test frequency. At first sight, this appears to contradict the findings of King et al. (2014), who reported similar correlations of IPD thresholds with audiometric threshold (*r* = 0.42) and with age (*r* = 0.45). However, as described in the *Introduction* section, their sample of listeners was characterized by a similar incidence of hearing impairment for the young and older listeners, and the correlation between age and the absolute threshold at low frequencies was not significant. Their young listeners with hearing loss might have had a form of hearing pathology that is not common in the general population and that adversely affects binaural sensitivity to TFS.

In the present study, the percentage of variance accounted for by the combination of audiometric threshold and age was relatively small (being largest at 42% for the 750-Hz test frequency). Thus, there is substantial individual variability in binaural TFS sensitivity that is not accounted for by audiometric threshold and age. This may partly reflect the fact that performance of the TFS-LF test (and other tests) is influenced by factors other than binaural TFS sensitivity per se. For example, some cognitive abilities may play a role, such as maintaining attention, using limited sensory information, and remembering “what to listen for” (e.g., Füllgrabe et al., 2015; Wallaert, Moore, & Lorenzi, 2016; Whiteford, Kreft, & Oxenham, 2017), while others, such as nonverbal fluid reasoning, do not seem to affect binaural TFS processing (Füllgrabe et al., 2018).

The absolute variability in *d* ′ scores decreased with increasing hearing loss and increasing age. However, this may be largely a consequence of the fact that the “best” performance for a given hearing loss and age tended to decrease with increasing hearing loss and age, while the worst performance could not drop below *d* ′ = 0. The relative variability, expressed as the coefficient of variation, actually increased with increasing audiometric threshold and increasing age. The limitation of the TFS-LF test that some listeners cannot perform the task at all can be largely overcome by use of the TFS-AF test (where AF stands for adaptive frequency), which gives an estimate of the highest frequency at which an IPD of ϕ (usually 180°) can be distinguished from an IPD of 0° (Füllgrabe et al., 2017; Füllgrabe & Moore, 2017). The great majority of listeners are able to perform the TFS-AF task to some extent, giving a graded measure of performance. Results for the TFS-AF test show that both absolute and relative variability across listeners tend to increase with increasing age (Füllgrabe et al., 2018).

The results for the test frequency of 500 Hz, for which the number of listeners was largest, suggested that *d* ′ scores did not change markedly with age up to about 40 years, but declined progressively thereafter. This pattern of results is similar to that inferred from a study using the TFS-AF test (Füllgrabe et al., 2018), although that study did not include middle-aged listeners. The deviation of the running average (thick black line in the right column of Figure 2) from the linear regression line fitted to the individual data over the whole range of ages may be a result of random variability or a sampling bias. However, over the age range 30 to 40 years, the running average was always based on a relatively large number of data points (at least 42), and the mean audiometric threshold of 7 dB HL for this age range was not better than that observed for younger adults (5 dB HL). Therefore, binaural TFS sensitivity might actually remain roughly constant with increasing age from 18 to 40 years, but declines with increasing age above 40 years.

## Conclusions

A meta-analysis was performed of data from 19 studies using the TFS-LF test to assess binaural sensitivity to TFS. The studies were conducted using listeners with a wide range of ages and audiometric thresholds at the test frequency (250, 500, 750, and 850 Hz). At least for the test frequency of 500 Hz, the distribution of audiometric thresholds within each age group was similar to that for the general population. Within this population-representative context of restricted audiometric variability in young and middle-aged adulthood and larger audiometric variability in older adulthood, binaural TFS sensitivity declined with increasing age across the adult lifespan and with increasing hearing loss beyond midlife. For all test frequencies, both audiometric threshold and age were significantly negatively correlated with TFS-LF sensitivity (with *r* varying from −0.19 to −0.64), but the correlation was always significantly higher for age than for audiometric threshold. Regression analyses showed that the standardized regression coefficient was greater for age than for audiometric threshold, and that there was a significant interaction. However, in combination, audiometric threshold and age only accounted for up to 42% of the variance in binaural TFS sensitivity, leaving a substantial amount of variance to be explained by other factors, such as cognitive abilities.
